# New insights on the cavitation development and the role of bubbles in Micro-Venturi channels^☆^

**DOI:** 10.1016/j.ultsonch.2025.107669

**Published:** 2025-11-07

**Authors:** Mohammadamin Maleki, Abhinav Priyadarshi, Jolyon Cleaves, Erçil Toyran, Ali Kosar, Iakovos Tzanakis, Morteza Ghorbani

**Affiliations:** a Sabanci University Nanotechnology Research and Application Center, 34956 Tuzla, Istanbul, Turkey; b Faculty of Engineering and Natural Science, Sabanci University, 34956 Tuzla, Istanbul, Turkey; c Faculty of Technology, Design and Environment, Oxford Brookes University, Headington, Oxford, OX3 0BP Wheatley, Oxford OX33 1HX, UK; d Specialised Imaging, Unit 32, Silk Mill Ind. Est. Brook Street, Tring, Hertfordshire, UK; e Center of Excellence for Functional Surfaces and Interfaces for Nano-Diagnostics (EFSUN), Sabancı University, Orhanli, 34956 Tuzla, Istanbul, Turkey; f Department of Materials, University of Oxford, Parks Road, Oxford OX1 3PH, UK

**Keywords:** Microfluidics, Hydrodynamic Cavitation, Micro-Venturi, Bubble Dynamics

## Abstract

This study investigates hydrodynamic cavitation (HC) inception and development in micro-Venturi channels, focusing on the mechanism that drives spatially irregular cavitation events. The study reveals that cavitation regeneration is primarily governed by the interaction between residual cavitation nuclei and low-pressure vortices. Using ultra-high-speed imaging and advanced bubble dynamics analysis, it was revealed that the residual nuclei trapped in the boundary layer or reverse flow near the sidewall of the microscale reactor are the key to cavitation regeneration. Their interaction with vortices shed from the shear layer, triggering spatially distributed inception events throughout the channel. Bubble velocity analysis showed a size-dependent pattern: smaller residual bubbles migrate upstream (negative velocities) before growing and being advected downstream (positive velocities), directly linking their motion dynamics to cavitation inception. Spectral analysis of the bubble populations demonstrated two frequency components: low-frequency signals in the transient regime, reflecting slow periodic replenishment of residual nuclei and high-frequency fluctuations driven by vortex activity. With increasing upstream pressure, the system shifted to periodic attached cavitation, characterized by regular shedding-driven fluctuations in bubble content. At even higher pressures, fully developed cavitation emerged, marked by intense shear-layer activity, sporadic downstream variations, and shear-induced bubble breakup that sustained mainstream cavitation. The findings of this study illustrate how residual nuclei and their interaction with transient vortices play a pivotal role in microscale cavitation inception, thereby offering critical insights on controlling cavitation in microfluidic systems such as “HC on a chip” reactors, where stochastic nucleation and bubble transport significantly influence performance.

## Graphical abstract

**Figure:**
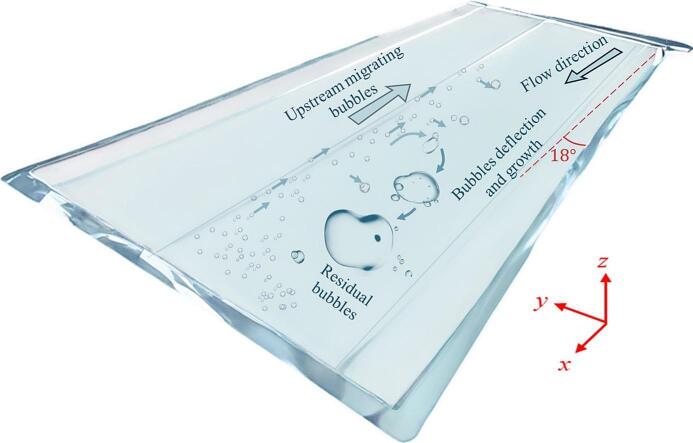

## 1 Introduction

Hydrodynamic cavitation (HC) is a phenomenon characterized by the formation, expansion, and subsequent collapse of vapor or gas bubbles, driven by localized pressure fluctuations within a flowing liquid. This process has been extensively studied at conventional scale and has been found to deteriorate the performance of hydraulic and turbomachinery[1]. However, during recent years, the field of microfluidic systems has flourished with the development of various microscale HC reactors. The growing interest in this microscale approach is due to its considerable advantages, such as enabling more robust control and precise monitoring of HC phenomenon. Additionally, recent studies[2,3] highlight the efficiency and improved performance of microscale HC, reinforcing its potential as an effective reactor platform.

Prior research[[4], [5], [6]] demonstrated that a reduction in the channel size leads to new flow physics. In a study by Mishra et al.[7] on micro-orifice flows, a remarkable scale effect on HC flow patterns was reported. They observed that the flow exhibited unique behavior, with the formation of a single stationary cavity downstream of the micro-orifice leading to flow choking. This behavior was fundamentally different from macroscale HC, where multiple factors including velocity and pressure scales influence the choking phenomenon. At the microscale, inception and bubble dynamics are significantly influenced by increased viscous and surface tension effects, along with surface roughness, nuclei population, residence time limitations, material properties and shear rates as demonstrated in studies on micro-orifices and micro-diaphragms; resulting in notable deviations from conventional scale cavitation behavior [8,9]. This fundamental shift in governing mechanisms necessitates a distinct understanding of HC physics at each scale, rather than relying on direct extrapolation from conventional to microscale conditions. The reduced residence time for nuclei growth and altered nuclei distribution patterns at the microscale contribute to substantially different cavitation dynamics [10]. Moreover, surface energy characteristics of silicon (Si), the primary material used in microfluidic devices, create hydrophobic and hydrophilic effects that significantly influence HC inception[11]. These effects are so pronounced that scaling laws cannot be directly applied to microdevice design.

Compared to studies on micro-orifices[7], studies specifically targeting HC in micro-Venturi geometries are relatively scarce. Micro-Venturi devices have a wide range of potential applications across various scientific and industrial fields. They can be effectively used as flow meters in two-phase systems, such as those found in the oil and gas industries[12]. Notable advantages of these micro-Venturi gas-oil flow meters include a wide flow range, minimal pressure loss, and high measurement precision and reliability[12]. Another important application of micro-Venturi devices is their use as mixers and injectors. As previous studies[13] have shown, these devices offer significant advantages over other injectors, such as eliminating the need for additional components for flow adjustment in the case of parallel injectors. The integration of microfluidic functionalities into conventional silicon MEMS devices has also garnered increasing interest, particularly with new technologies in biology and life sciences. Due to the increasing complexity of devices, the parallel operation of multiple flow generators is sometimes required. While standard microfluidic components can be easily parallelized, the miniaturization of effective pumps for flow or pressure generation remains a challenge[13]. Furthermore, these micro-Venturi devices can be implemented as components in sensors and microfluidic systems, as demonstrated by Cheri et al. in their study on an optofluidic flow sensor[14]. Micro-Venturi devices can also serve as a tool in environmental engineering for applications like wastewater treatment and advanced oxidation processes. In a recent study by Maleki et al.[15], the chemical effects of micro-scale hydrodynamic cavitation (HC) bubble collapse were investigated using salicylic acid (SA) dosimetry. Different HC micro-reactors, including a micro-Venturi, were utilized, and a significant improvement in SA production was observed in the micro-devices. This finding highlights the high potential of micro-Venturi devices in wastewater treatment and oxidation process applications. These devices can be utilized with a wide variety of single-phase and multi-phase fluids, including gases, oils, water, and biofluids. Depending on the fluid type, they can be operated across a wide range of temperatures, pressures, and flow rates. Limited existing studies[16] unveiled phenomena such as the formation of a single elongated vapor cavity along the micro-Venturi centerline, which is in contrast to the dense bubble population observed near walls at the macroscale Venturi. Also, micro-Venturi flows can maintain exceptionally high-tension levels without exhibiting the choking behavior typical of larger scales. Interestingly, the possible presence of Kelvin-Helmholtz (K-H) instabilities in cavitating flows was observed, suggesting they may influence HC inception or even consistent with the overall cloud shedding behavior. However, a thorough investigation explicitly exploring the role of K-H instabilities as a potential HC shedding mechanism in fully developed cavitating micro-flows is yet to be conducted.

A pivotal aspect of HC at the micro-domains is the inception, which hinges on the presence of microscopic weak spots—nuclei containing traces of noncondensable gas. Without such gas nuclei, working fluids such as water can withstand tensile stresses exceeding several tens of MPa due to their metastable state, delaying potential homogenous cavitation to extremely low pressures[17]. Critically, related nucleation studies elucidated the origin and behavior of HC nuclei—bubbles or particles that enable liquid rupture under reduced negative pressures[18]. Gas nuclei within the liquid can be energized and grow in low-pressure regions, where gas diffusion can accelerate bubbles growth at early stages.

Several studies explored the influence of nuclei bubbles on HC[19], including the impact of the initial bubble size on HC bubble growth and breakup in a Venturi channel[20], the effects of the injection location and dissolved gas on HC on hydrofoils[21], and the influence of the injection rate on inception event probability in a backward-facing step[22]. More recently, the role of residual bubbles from HC bubble collapse in sustaining attached sheet HC in a nozzle configuration was investigated for varying surface curvatures (and thus pressure gradients)[23]. Bubble tracking revealed that both thick boundary layers and separation facilitated upstream microbubble migration, with diffusion promoting growth. These bubbles, upon reaching boundary layer size in a fixed location downstream of the minimum pressure region, were either swept away or initiated new HC events.

While the importance of residual microbubbles has been recognized in the literature[23,24], there are few studies on investigating their influence on sustaining HC. Studies addressing the transition between HC flow regimes (e.g., detached, periodic, fully developed) within shallow micro-Venturi configurations are limited[20]. Notably, no study has yet explored the interaction between residual bubbles and shedding vortices within the shear layer, or how this interaction influences the transient cavitation flow regime. This work bridges that gap by investigating HC inception and regime evolution in a micro-Venturi channel through ultra-high-speed imaging and bubble tracking. We demonstrate that residual microbubbles, trapped in boundary layers or reverse flows, sustain HC in low-pressure regions by interacting with transient shear-layer vortices. By linking bubble dynamics (e.g., size-dependent migration, dominant frequencies) to microscale flow structures (e.g., shear-layer vortices), we reveal how bubble-vortex interactions and local pressure gradients dictate regime transitions. The findings of this study will contribute to advances on the design of microfluidic devices, offering strategies to control HC bubble nucleation through geometry optimization or flow conditioning, thereby mitigating HC-induced damage in applications ranging from biomedical sonication to microreactors.

## 2 Methodology

### 2.1 Design and fabrication

A microscale HC reactor, (the micro-Venturi channel), was designed with a compact footprint of 14 × 6 mm^2^. The reactor consists of two main sections: inlet and outlet channels having a uniform depth of 60 µm. The inlet and outlet ports, each having a diameter of 900 µm, are spaced 8900 µm apart and are connected to a sandwich holder for fluid delivery and discharge. The details of the micro-venturi device geometry are included in Table 1. The reactor was constructed according to the semiconductor-based microfabrication techniques from two layers, Si and glass with the corresponding channels and ports etched into the Si layer. The details about the fabrication technique can be found elsewhere[25,26].

**Table 1:** micro-venturi device geometry details.

Throat Width (μm)	Channel Height (μm)	Throat Length (μm)	Converging Angle (°)	Diverging Angle (°)	Inlet Width (μm)	Contraction Ratio (μm)	Inlet Hydraulic Diameter (μm)	Throat Hydraulic Diameter (μm)
100	60	0	4	4	658	6.58	110	150

### 2.2 Experimental setup and Procedure

**Fig. 1:**
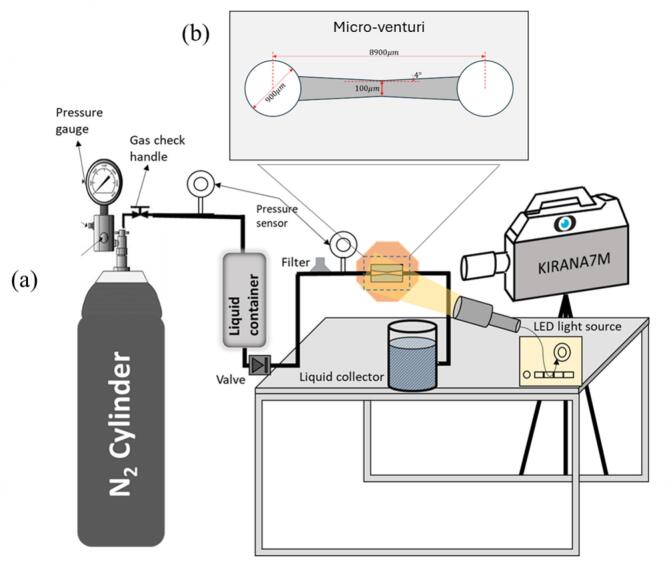
Schematic of the hydrodynamic cavitation reactor experimental setup used for high-speed visualization in the micro-venturi configuration. (a) test rig, and (b) micro-venturi device.

Within the experimental test rig (Fig. 1(a)), a pressurized container of pure nitrogen gas (BOC, UK), a sample container for the liquid (Swagelok, Italy), multiple sensors to measure the pressures (Omega, UK, with an accuracy of ± 0.25 % and measuring up to 3000 psi (absolute uncertainty of ± 7.5 psi (51.71 kPa)), which leads to maximum uncertainty of 0.0475 bar under operation condition in this study), stainless steel tubing (Swagelok, Italy), and a sandwich holder were employed. The reactor (Fig. 1(b)) was placed in the holder and had connections for the liquid to enter and leave. The reactor was then tightly sealed via micro-O-rings with transparent Poly (methacrylic acid methyl ester) (PMMA) lids for safety. To start with the experiment, the container was filled with clean de-ionized water, and the nitrogen gas was slowly released into the system to push water through. The working fluid was held at a constant room temperature (20 °C) during experiments. The storage tank was filled with de-ionized water at room conditions. The effect of N_2_ Pressurization on dissolved gas content is assumed to be negligible within the short experimental time frame as the residence time of the fluid under pressure in the supply line is very short. Moreover, based on Henry's Law constant (1600 atm.lit/mol for N_2_), the N_2_ diffusion in water required to significantly alter the baseline air saturation level is extremely slow relative to the flow rate. The system was operated only after establishing stability within the observed patterns with continuous monitoring of the channel and that of the pressure regulator. The water temperature was also held stable at room temperature. The water was filtered by a 15 µm nominal pore size micro-T-type filter (Swagelok, Italy) to remove any unwanted particles before entering the reactor. The pressure of the gas before the holder was monitored with a pressure sensor. By changing the upstream pressure (pressure of the gas), different cavitating flow patterns were obtained, while the pressure after the reactor was kept at normal atmospheric pressure. The influence of three distinct flow regimes (detached, periodic, fully developed), achieved by varying upstream pressure to 14.5 bar (P1), 17 bar (P2), and 19 bar (P3) (measured as gauge pressures), on cavitation characteristics was investigated at constant room temperature. The system was operated only after establishing stability within the observed patterns with continuous monitoring of the channel and that of the pressure regulator. The water temperature was also held stable at room temperature. While the present study establishes the critical fluid dynamics and spectral characteristics across three distinct cavitation regimes, the next logical step involves a rigorous mapping of the regime boundaries. Future work will focus on performing comprehensive pressure or cavitation number (σ) sweep cycles (ramp-up and ramp-down) to assess the degree of hysteresis in regime transitions. This effort will culminate in a validated regime diagram, complete with quantified error bands, providing a robust, generalized tool for predicting the operational stability of the micro-venturi device. Table 2 provides details on the cavitation type and Reynolds number associated with each flow condition. Reynolds ($Re_{D}=UD_{h}/\nu$) and cavitation numbers ($\sigma =\left(p_{\mathrm{in}}-p_{\mathrm{sat}}\right)/\left(\frac{1}{2}\rho_{w}U^{2}\right)$ were calculated using the mean velocity (U) and hydraulic diameter ($D_{h}$) of the constriction region. Additionally, the cavitation number was determined based on the upstream pressure ($p_{\mathrm{in}}$).

**Table 2:** Flow conditions and dimensionless numbers.

Parameters	P1 Flow	P2 Flow	P3 Flow
Upstream gauge pressure (bar)	14.5	17	19
Downstream pressure (bar)	0.987	0.987	0.987
Mean velocity at throat (m/s)	19.17	24.71	36.01
Cavitation Number (−)	0.75	0.55	0.29
Reynolds Number (−)	5,340	5,955	7,530
Volumetric flow rates (ml/s)	0.115	0.148	0.216

*Re* numbers ($Re_{D}=UD_{h}/\nu$) is based on the hydraulic diameter ($D_{h})$, U is the mean velocity within the microchannel calculated based on the volumetric flow rate *(V) and the channel cross-section at constriction (Ac)*, and ν is kinematic viscosity) and cavitation numbers ($\sigma ={(p}_{\mathrm{in}}-p_{\mathrm{sat}})/\left(\frac{1}{2}\rho_{w}U^{2}\right)$ where defined based on inlet pressure, $p_{\mathrm{in}}$ ($p_{\mathrm{sat}},\rho_{w}$ and ν represent water saturation pressure, water density and kinematic viscosity, respectively).

Cavitation inception and dynamics within the microscale HC reactor were visualized with a KIRANA7M (Specialised Imaging Ltd., Pitstone, UK) ultra-high-speed video camera equipped with a Navitar 12X zoom lens with 2X f-mount adaptor and 2X lens attachment. The camera has a resolution of 924 x 768 pixels with 200 ns exposure and depth of focus ranging from between 0.35 mm and 0.01 mm depending on the zoom setting. The system was meticulously calibrated to yield an effective pixel size (spatial resolution) of 1.34  μm/px, resulting in a field of view of approximately 1238  μm × 1029  μm. The depth of field for the system, depending on the zoom setting used, was estimated to range between 10  μm and 350  μm, ensuring all phenomena within the test region remained within focus. The data presented throughout this study were acquired from a positioned Region of Interest (ROI) of 840 × 280 pixels. This cropped frame size maintains the calibrated spatial resolution of 1.34  μm/px, which was used for all subsequent conversions of dimensional parameters and velocity components to SI units (m and m/s). Illumination was provided by a collimated bright light with 370 J flash head and 750 µs pulse duration (SI-MSFH-370). The visualization were taken at frame rates up to 2 million frames per second (fps). The reactor was illuminated additionally using a high-power LED light source (Karl Storz Power LED 175, Germany) and the images were analyzed to understand the behavior of the flow inside the reactor. To ensure consistency in the analysis, the camera settings for brightness and contrast were kept the same for all the recordings.

Roughness on the channel surface is a critical factor in cavitating flows, as it provides active nucleation sites that sustain gas nuclei derived from gas diffusion, or residual gas remaining after bubble collapses.

**Fig. 2:**
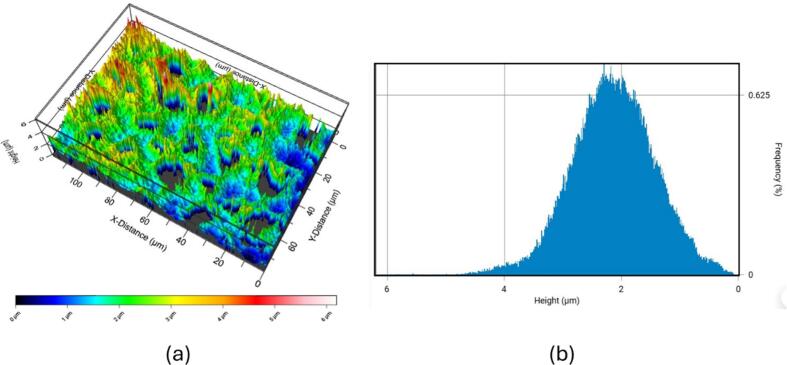
Laser confocal scanning microscopic images of the silicon surface (a) 3D distribution representation of the silicon surface with contours indicating the relative height of the surface (b) frequency of surface heigh on the silicon surface.

The silicon surface was characterized via Laser Confocal Scanning Microscopy, as shown in Fig. 2. The 3D image (Fig. 2(a)) details the surface morphology, while the height frequency distribution (Fig. 2(b)) confirms a near-Gaussian profile. This distribution is centered around a mean height of approximately 2µ, with the standard deviation (Root Mean Square Roughness, Rq) measured at about 1µ.

**Fig. 3:**
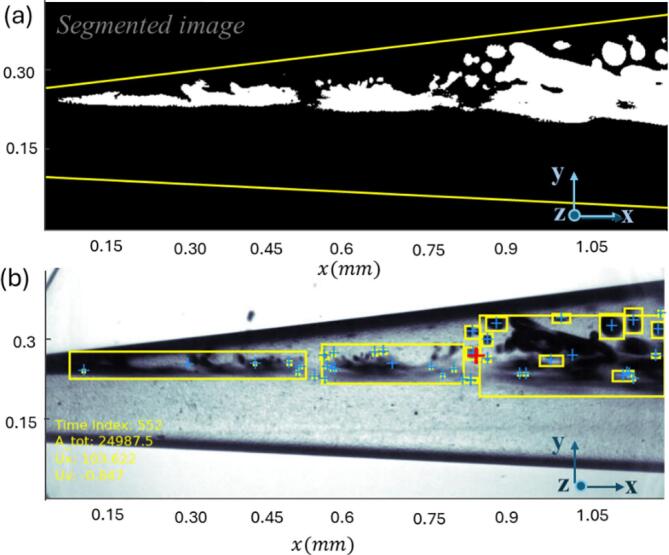
(a) Segmented image obtained using the GMM technique, where the foreground regions are represented in white and the background in black. (b) Detected bubbles, enclosed in yellow boxes, with their centroids indicated by blue plus signs for further analysis.

Bubble dynamics were analyzed using the technique presented in Fig. 3, with the complete methodology described in Appendix A.

## 3 Results and discussion

### 3.1 Micro-Venturi cavitating flow development

**Fig. 4:**
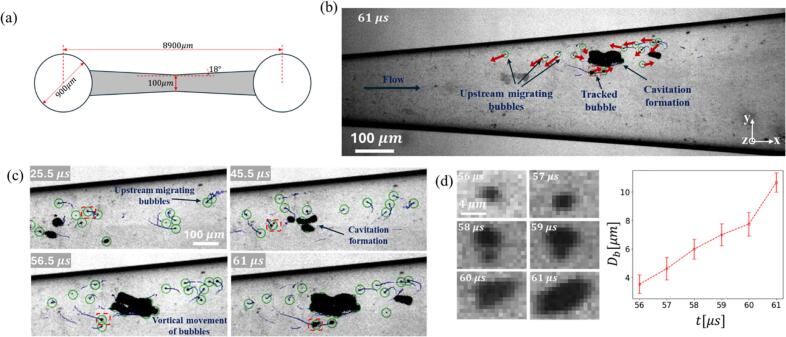
Characterization of Bubble Behavior in a Microfluidic Venturi (P1 cavitation regime). (a) Schematic of the micro-venturi geometry with all relevant dimensions. (b) Depiction of the trajectories of bubbles as they migrate upstream near the bottom and side walls of the channel. (c) A detailed trajectory of a single, selected bubble (within the red box), captured at successive time intervals between 25.5 µs and 61 µs. (d) A close-up view of the bubble's final growth stage, including a corresponding plot of its diameter versus time for the interval between 56 µs and 61 µs. Diameters were calculated based on a pixel threshold of 0.7, and the error bars show the variability of the diameter measurements when the threshold was varied between 0.6 and 0.8. These smaller bubbles then coalesce with the main cavity before its subsequent implosion (multimedia available online).

Fig. 4 (multimedia available online) shows bubble trajectories within the shallow micro-Venturi channel captured at 2 million fps under an upstream pressure of 14.5 bar. The trajectory lines in this case were represented using ImageJ software [27]. Individual bubbles, ranging from 1.3 to 20 µm and highlighted with green circles, were tracked over the preceding 21-time steps (10.5 µs) with their paths depicted as dark blue lines. Bubbles near the sidewall exhibited slow upstream migration, evident from the shorter path lines in Fig. 4(a) indicating their residence within the boundary layer. In contrast larger bubbles located in the central region of the channel moved downstream more rapidly. Fig. 4 further reveals that these upstream-migrating bubbles, at certain locations along the sidewall, were deflected towards the center of the channel, where they grew and accelerated due to higher flow velocities. Their curved trajectories suggest entrainment within spanwise vortices. Fig. 4(b) provides the details of a single bubble growth (in a red dashed box) and displacement over time. The directions of the red flashes track the movement of bubbles within the channels, revealing a pronounced tendency for bubbles located near the channel walls to move in a direction opposing the mainstream flow. Moreover, the dark blue pathlines provide a secondary means of roughly predicting the direction of movement. They show the bubble's history over the last five snapshots, allowing us to estimate its speed and direction. Initially moving slowly near the sidewall (25.5–45.5 µs), the bubble then migrated toward the centerline (45.5–56.5 µs), followed by rapid growth and acceleration (56.5–61 µs). Fig. 4(c) provides a detailed step-by-step analysis of the bubble growth (between 57 µs and 61 µs) showing that bubble’s size nearly tripled within 5 µs, indicating exposure to a local pressure drop likely originated by vortices generated from the velocity gradient between reverse and core flows.

**Fig. 5:**
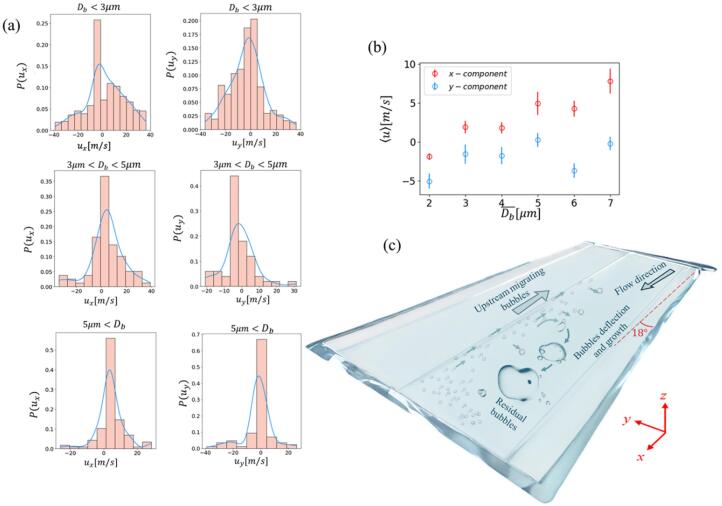
(a) Bubbles velocity distribution for various size ranges. (b) Ensemble mean velocity as a function of mean bubble size (${\overset{¯}{D}}_{b}$ represents bubble diameter ranges and of ± 0.5 μm error bars represent standard deviation of bubble velocities in each group). (c) Schematic diagram of the cavitation mechanism at an upstream pressure of 14.6 bar.

Fig. 5 complements the bubble tracking analysis of Fig. 4 by quantifying bubble growth and velocity distributions (u_x_ and u_y_ are corresponding to the streamwise and cross streamwise velocity components along the x and y axis in Fig. 4(b)). In specific, Fig. 5(a) shows the probability distributions of the bubble velocity components in both streamwise ($u_{x}$) and cross-flow ($u_{y}$) directions, categorized by bubble size: $D_{b}$ < 3 µm, 3 µm< $D_{b}$ < 5 µm and 5 µm < $D_{b}$. For the smallest bubbles ($D_{b}$ < 3 µm), approximately 25 % had negative streamwise velocities between −6 m/s and 0 m/s, indicating slow upstream migration near the bottom surface and sidewalls (Fig. 4) contributing significantly to the regeneration of cavitation at various locations within the channel.

While the 3D structure could not be measured directly, the relationship between bubble velocity and size offers clues about their shape. Data from Fig. 4, Fig. 5 show that for bubbles smaller than the channel depth, velocity increases with bubble size. Assuming these small bubbles move passively with the flow (with negligible slip at the liquid-bubble interface), we can deduce that they are located in low-momentum regions near the boundary layers, which is consistent with a three-dimensional near spherical shape [20,23]. The continued growth and accelerating velocity of these bubbles suggest they are moving toward the high-velocity region at the center of the channel. Due to the small depth of the channel, it is a reasonable assumption to ignore the superposition of bubbles.

We hypothesized that these microbubbles mostly form from the condensation of vapor cavities, leaving behind residual air that resists redissolution even under high pressures, consistent with observations in the Podbevsek et al. study [5]. The existence of two types of active nucleation sites—stable surface nuclei and transient residual microbubbles—is highly probable in this micro-channel flow. Our Laser Confocal Scanning Microscopy analysis (Fig. 2) reveals a significant surface roughness (Rq ≈1 μm) which is capable of sustaining stable gas pockets when wetted, thus providing the necessary initial condition for inception. However, evidence suggests that the residual microbubbles resulting from previous collapse events also contribute substantially to the high frequency of re-inception. Two key observations suggest that the majority are remnants of downstream bubble collapses. Firstly, these microbubbles are present in downstream regions where collapses occur and exhibit regrowth after being carried upstream into low-pressure zones. Secondly, our spectral analysis in the following section demonstrates that the appearance and disappearance of these microbubbles are synchronized with larger bubbles. This contrasts with the expected persistent presence of microbubbles generated from surface or free nuclei. Fig. 5(b) illustrates that larger bubbles tend to have a larger proportion of positive streamwise velocities at an increased rate attributed to increased interaction with the central flow. (75 %, 88 %, and 96 % positive $u_{x}$ for $D_{b}$ < 3 µm, 3 µm< $D_{b}$ < 5 µm and 5 µm< $D_{b}$, respectively). The cross-flow velocities ($u_{y}$) were predominantly negative for smaller diameters ($D_{b}$ < 5 µm) due to upstream movement along converging walls, also observed in prior studies (Ram et al. 2020) of attached cavitation over a curved hydrofoil. Two key mechanisms have been proposed to explain this upstream migration: (i) a strong adverse pressure gradient (APG) combined with thick boundary layer pushing small bubbles upstream within low-momentum zones, and (ii) reverse induced by flow separation when the APG is strong enough. Under condition (i), a thick, low-momentum boundary layer forms near the wall surface. While the bulk flow velocity remains positive, the small bubbles trapped within the lowest-momentum zone of the boundary layer can be pushed upstream due to the force exerted by the APG. In this non-separated boundary layer, the bubbles are driven upstream primarily by the APG. When the flow separates, as in condition (ii), the APG is often reduced or eliminated near the separation point. Within the recirculating reverse-flow zone characteristic of separation, the bubbles are driven upstream primarily by the fluid drag imposed by the reverse flow. Both mechanisms result in the observed slow upstream migration of bubbles, which typically lasts for a few milliseconds. Within transient low-pressure regions—likely spanwise vortices formed in shear layers—bubbles grew before collapsing downstream upon pressure recovery. This collapse regenerated residual bubbles, driving repeated cavitation cycles along the channel, as seen in the pathlines of Fig. 4, Fig. 4. In this study, lateral channel expansion generated such an APG, driving flow separation at the sidewalls and generating reverse flows that transported bubbles upstream along sidewalls, as schematically illustrated in Fig. 5(c). Further from the sidewalls, a combination of APG and low momentum zones near the bottom surface aided the upstream migration of small bubbles towards the flow centreline. These residual bubbles (visible in the microbubble pathlines shown in Fig. 4, Fig. 4) expanded within transient low-pressure zones, likely corresponding to vortices formed in the shear layer[28] (Fig. 5). These vortices transport the growing bubbles further downstream. Upon pressure recovery within these vortices, the bubbles collapsed, forming new residual bubbles that triggered repeated cavitation cycles along the channel. This cyclical process sustained repeated cavitation events along the channel length, consistent with observations in Fig. 4. These observations suggest a key difference between macroscale and shallow micro-Venturi configurations[23]. Unlike macroscale systems, where residual bubbles are trapped and grow at a fixed location downstream of the nozzle throat, the shallow micro-Venturi's residual bubbles serve as nucleation points. These bubbles initiate new cavitation events when exposed to the transient, localized low-pressure zones produced by shedding vortices. This process results in a transient cavitation regime characterized by random initiation sites along the channel.

### 3.2 Statistical Analysis, Tracking, and characterization of HC bubbles under different

#### 3.2.1 Flow conditions

In this section, we conducted statistical analysis of cavitating bubbles under three distinct flow conditions, at 14.5 bar (P1 flow), 17 bar (P2 flow), and 19 bar (P3 flow), to evaluate how varying upstream pressures affect cavitation characteristics within the micro-Venturi.

**Fig. 6:**
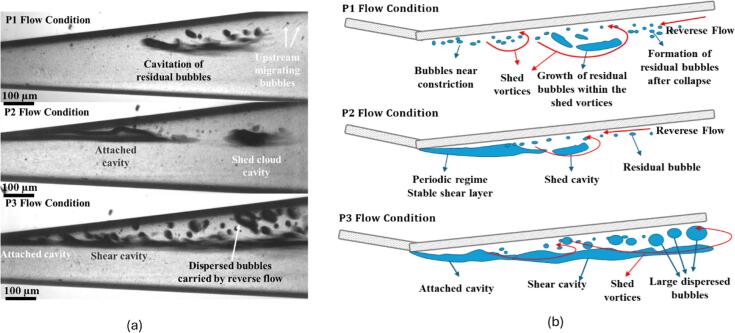
Cavitation patterns for 3 different upstream pressures of (a)14.5 bar (multimedia available online), (b) 17 bar (multimedia available online) and, (c) 19 bar (multimedia available online). Schematic corresponding to (d) P1, (e) P2, and (f) P3 flow regimes.

Flow conditions strongly influence both cavitation inception and bubble breakup mechanisms. As previously discussed, under P1 flow, high-temporal resolution imaging (2 million fps) revealed transient cavitation near the bottom wall. Fig. 6(a) (multimedia available online), 6(b) (multimedia available online) and 6(c) (multimedia available online) show representative high-speed images (captured at 80,000 fps) for the three conditions. A key challenge was that a high temporal resolution, necessary for detailed analysis, could only be maintained for a very short period (approximately 200 sequential snapshots). This prevented a long-term statistical study of bubble behavior across different cavitation regimes. To address this, a lower temporal resolution of 80,000 fps was adopted for statistical analysis. This allowed for a much longer observation period, sufficient to capture the dominant frequencies of bubble motion within the cavitating flow. Under P1 flow conditions, transient local cavitation formed on the bottom surface and sidewall of the channel. This transient regime is characterized by cavitation generated sporadically in both space and time, with detached cavities forming and disappearing throughout the channel. These cavities typically form within the shed vortices of the shear layer. As the upstream pressure increased to P2, an attached sheet cavitation developed near the sidewall at the constriction, which periodically shed as a cloud cavitation. Under a sufficiently strong APG and velocity gradient, the pressure drop within the shear layer becomes low enough to sustain a cavity attached to the throat edge. This attached cavity sheds regularly due to a re-entrant jet or downstream collapses, a regime known as periodic cavitation. Further increasing the upstream pressure to P3 enhanced the attached cavitation and intensified the shear layer cavitation, where a steep velocity gradient triggered shear-induced bubble breakup. When the shear layer is fully occupied by the vapor phase, the flow is considered a fully developed cavitation.

The resulting bubbles, which were significantly larger than the residual bubbles post-collapse, predominantly entrained and moved upstream with the reverse flow. Across all flow regimes, cavitation pattern exhibited asymmetry, occurring mainly on the top sidewall due to the flow pattern within the micro-Venturi channel. High velocities through the constriction and flow separation at the diverging section generated a low-pressure zone near the lower wall and a separated flow region near the upper wall. This imbalance, compounded by outlet perturbations, prevented the flow from switching sides, as shown in prior work[29].

The formation of attached sheet cavitation near the constriction required sufficiently high velocities in the convergent section to induce a pressure drop triggering phase transition at the diverging edge. Prior studies[21] linked this process to sparse streaks of cavitating spanwise vortices anchored to the leading edge of the diverging section. A decrease in the cavitation number from 0.75 (P1) (Fig. 6(d)) to 0.55 (P2) (Fig. 6(e)) shifts the regime from detached to the attached cavitation. This regime is associated with periodic shedding of vapor clouds occurred (Fig. 6(e)). While direct evidence is lacking in this study, previous work[23] suggested that residual bubbles contribute to attached cavity sustenance, implying that both free-stream and residual nuclei promote cavity growth and detachment. Under the P3 regime, cavitation sharply intensified within the shear layer downstream of the attached cavitation (Fig. 6(f)). Increased upstream pressure and the resulting higher flow rate within the channel elevate shear stresses within the shear layer, as established in prior research[30,31]. These shear stresses in the shear layer fragmented the larger bubbles into secondary bubbles that were notably larger than the typical residual bubbles[21].

Two mechanisms contributed to bubble breakup downstream of the constriction. The first involves condensation and the formation of small residual bubbles (<3 µm), which persisted even at high pressures (the non-condensable gas component of the cavities formed these residual bubbles after collapse)[3]. Under low shear stress conditions (low upstream pressure, P1(Fig. 6, Fig. 6), this mechanism dominated, producing tiny secondary bubbles. These residuals migrated upstream through low-momentum regions within the bottom boundary layer, where they were trapped by transient low-pressure vortices[23,24]. Most of these bubbles concentrated near the bottom silicon wall rather than the top glass plate, likely due to rough surface of Si. The laser confocal scanning microscopic results showed that the distance between peak and valley over the silicon surface can be as large as 5μm providing susceptible zones for bubble entrapment. Their accumulation near the rough Si wall—rather than the smooth glass top—suggests that surface roughness increased boundary layer thickness and promoted nucleation site availability. The second breakup mechanism was driven by high shear stress[32,33] acting on larger bubbles (>7 µm), such as those within the shear layer, particularly under higher upstream pressures (P3 flow condition (Fig. 6, Fig. 6), and to some extent P2 flow condition (Fig. 6, Fig. 6). This shear induced fragmentation generated large bubbles that were then advected by the reverse flow, reinforcing the cavitation dynamics seen in Fig. 6, Fig. 6.

**Fig. 7:**
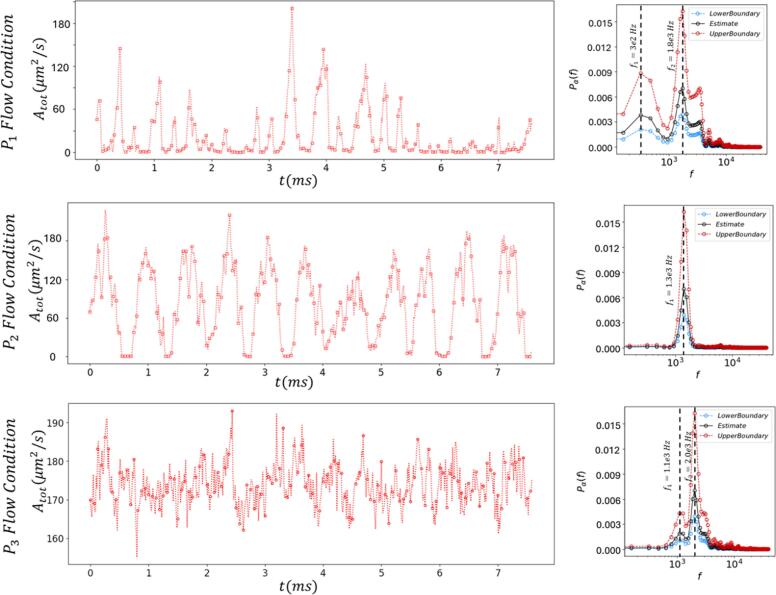
Variation in bubble number (the left column) and corresponding spectral content (the right column) over time for different upstream pressures (t=0 is the reference time). Lower and upper bounds are obtained based on confidence interval of 95 %.

**Fig. 8:**
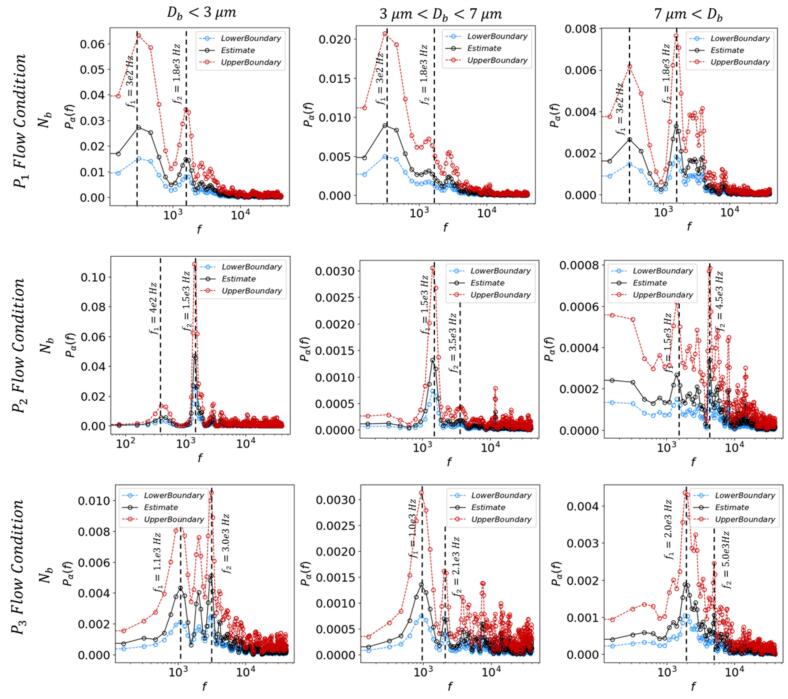
Spectral content over time for different upstream pressures and different bubble diameters. Lower and upper bounds are obtained based on confidence interval of 95%.

A statistical analysis of the cavitation bubbles was performed to evaluate the effects of each flow condition and breakup mechanism on bubble dynamics and cavitation. To ensure the robustness of the spectral estimates, the Welch's method was employed, and the resulting PSD plots (Fig. 7, Fig. 8) include upper and lower bounds of estimation using a confidence interval (95 % confidence). Fig. 7, Fig. 8 illustrated variations in the bubble count and total bubble area across flow conditions. Fig. 7 (left column) depicted bubble count variations across size ranges ($D_{b}$ < 3 µm, 3 µm< $D_{b}$ < 7 µm and 7 µm < $D_{b}$), while Fig. 7 (right column) presented the PSD for these ranges. In P1 flow condition, two dominant frequencies were observed across all size ranges: a low frequency at 3 × 10^2^ Hz and a high frequency at 1.8 × 10^3^ Hz. The proportion of small bubbles ($D_{b}$ < 3 µm) was the highest, while the number of bubbles in the 3 µm< $D_{b}$ < 7 µm and 7 µm < $D_{b}$ ranges were of the same order. This resulted in a higher PSD level for small bubbles compared to the other two size ranges. In the transient regime (P1), with an increase in the size of the bubbles the PSD level of low frequency peak decreases, indicating that the influence of low-frequency variations on smaller bubbles was more pronounced. The results show that the dominant frequencies of bubble motion are dependent on both bubble size and the specific flow regime. Consistent with our prior study[15], we found that in comparable micro-Venturi configurations, dominant motion frequencies ranging from 2 to 5.5 x 10^3^ Hz are associated with maximum void fraction fluctuations within the shear layer. This finding substantiates that these high dominant frequencies correspond to the vortex shedding mechanism within the shear layer.

As the upstream pressure increased (P2 and P3 flow conditions), the low-frequency peak gradually diminished, and new high-frequency fluctuations emerged around 4 × 10^3^ Hz. Residual bubbles migrated upstream slowly, repeatedly encountering shed vortices in the shear layer (which exhibited higher frequencies). These bubbles grew, transported, collapsed within the vortices, and producing new residual bubbles. This increase in residual bubbles can amplify vapor generation events and consequently the number of large bubbles. At low upstream pressures, when the flow field is depleted of residual bubbles, only free-stream nuclei can contribute to the generation of large bubbles within the core of transient low-pressure vortices. Free-stream nuclei are less frequent, and the probability of cavitation is lower when the flow field is depleted of residual bubbles. However, when cavitation does occur, it generates a new set of residual bubbles, which contributes to subsequent cavitation/collapse events and the regeneration of the residual bubble cycle until the field is once again depleted. In the transient regime, new cavitation events are initiated by the superposition of slow, upstream-moving residual bubbles with transient local low-pressure regions (shedding vortices). As long as a sufficient supply of residual bubbles is available, cavitation shedding occurs at the same frequency as vortex shedding. However, the channel can become depleted of residual bubbles, a process that may be caused by a regular increase in pressure from the self-sustained, low-frequency motions of the separation bubble[34]. This depletion may result from a regular increase in channel pressure caused by the low-frequency motions of the separation bubble, which prevents the formation of new cavitation events. This leads to a long gap before a new cavitation cascade begins, as shown in Fig. 7 (P1 Flows). A notable portion of residual bubbles from collapse events are swept downstream, with only a fraction near the boundaries being retained to sustain new cavitation. These results suggest that the population of residual bubbles is consistent by two distinct mechanisms: first, their high-frequency consumption and regeneration by shedding vortices, and second, a low-frequency depletion indirectly driven by variations in the channel pressure field. Under high shear stress conditions (shear breakup, P3 and to some extent P2 flow conditions), shear-induced bubbles continuously replenished mainstream cavitation, eliminating low-frequency bubble count (void fraction) variations. The continued presence of cavitation in the shear layer is likely due to the more significant pressure drop within this region. This pressure drop is a direct consequence of a stronger velocity gradient, which in turn is caused by an increased mean flow rate. This mechanism enhances the sustainability of cavitation within the low-pressure cores of the shedding vortices, thereby preventing a prolonged depletion of the channel's cavitation and residual bubble population. Total cavitation area ($A_{\mathrm{tot}}$) trends mirrored bubble count patterns (Fig. 8). In P1, both low (∼ 3 × 10^2^ Hz) and high (1.8 × 10^3^ Hz) frequency peaks appeared, with the high-frequency PSD dominating due to the greater contribution from large bubbles to the total area. In P2 flow condition, $A_{\mathrm{tot}}$ varied regularly with a single high-frequency peak, reflecting periodic cavitation. P3 flow condition displayed sporadic behavior with two high-frequency peaks. The data presented in Fig. 8 demonstrated that elevating upstream pressure significantly increased the extent of cavitation coverage within the channel, signifying cavitation intensification. Specifically, the P3 flow condition showed persistent, substantial cavitation occupancy. Conversely, the P2 and, especially, the P1 flow conditions displayed transient periods of minimal cavitation, with P1 exhibiting the most pronounced reduction.

**Fig. 9:**
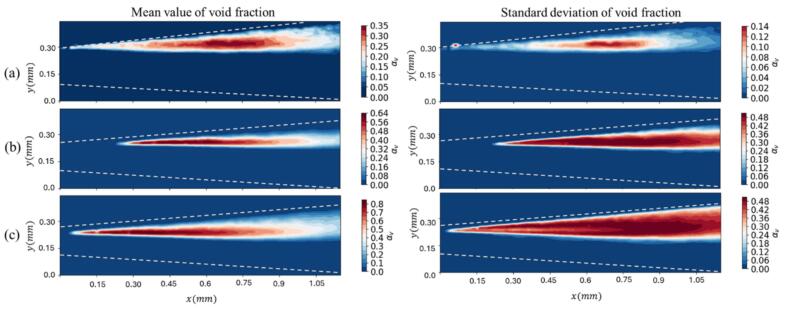
Contours of mean (left column) and standard deviation (right column) of mean void fractions for P1 to P3.

Mean and standard deviation contours of void fraction are provided in Fig. 9. A key observation across all regimes is the asymmetry in the formation of the cavitation within the channel. This asymmetry is demonstrated in the mean and standard deviation contours of the void fraction (Fig. 9). The persistent difference in the integrated void fraction distribution between the top and bottom sidewalls indicates that the cavitation sheet preferentially attaches and is sustained on one sidewall, which is consistent with results of section 3.2.

**Fig. 10:**
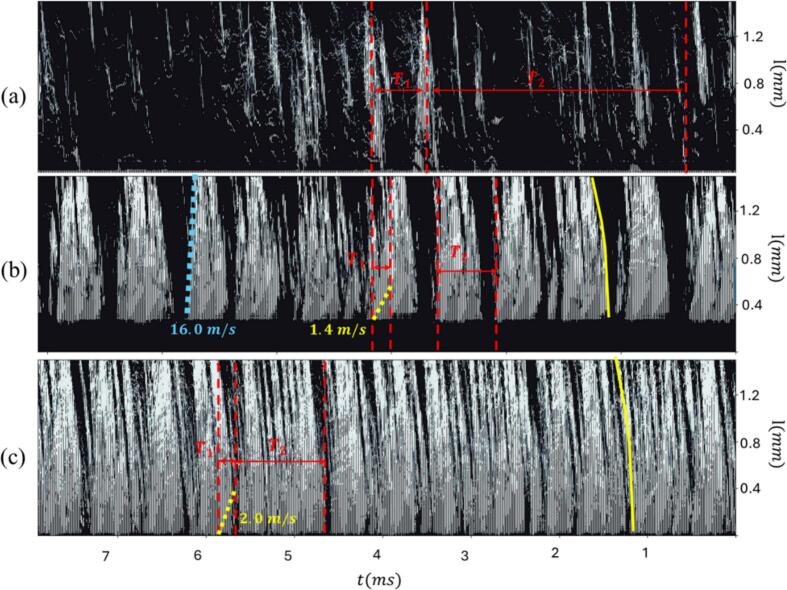
T-x diagram obtained by stacking 616 successive high-speed images of (a) P1 flow condition (T1 = 0.58 ms, T2 = 2.7 ms), (b) P2 flow condition (T1 = 0.37 ms, T2 = 0.71 ms), and (c) P3 flow condition (T1 = 0.28 ms, T2 = 0.92 ms). The solid yellow line denotes the overall cavitation cloud evolution. The dashed yellow line tracks the re-entrant jet progression along the channel wall, which dictates the cloud shedding cycle. The dashed blue line indicates the movement of the condensation front. The temporal points T1 and T2 are provided to illustrate moments corresponding to the high- and low-frequency dominant motions resolved by the velocity fluctuation analysis.

The flow periodicity was quantified using the Strouhal number (St), calculated following the definition provided in Reference[35]: St = f⋅L_c_/U_t_. Here, f represents the characteristic frequency, U_t_ is the mean flow velocity at the micro-venturi throat, and L_c_ is the mean cavity length. The characteristic length Lc was determined by analyzing the gradient of the mean void fraction distribution in both time and along the y-axis across the primary cavitation region (Fig. 9, Fig. 2). Furthermore, the flow dynamics were characterized using a Time-Distance (T − x) map (Fig. 10), generated by stacking sequential images. This map clearly visualizes the high- and low-frequency void fraction dynamics, providing characteristic curves associated with cavitation evolution, shedding via the re-entrant jet, and the condensation front progression.

Analysis of the Strouhal numbers (Table 3) alongside the T − x map (Fig. 10) allows for a clear physical linkage between the measured frequencies and the flow dynamics. For the P1 cavitation regime, the dominant low-frequency Strouhal number is associated with the time gap required for the reappearance of the next cascade of cavitation shedding after the channel briefly clears of void fraction. The high-frequency Strouhal number in this regime, conversely, is directly linked to the periodic cavitation shedding frequency. For the P2 cavitation regime, the T − x map reveals clear signatures of front condensation and re-entrant jet propagation, consistent with observations reported in the literature [36]. The characteristic slope of the condensation front, measured at approximately 16 m/s, is within the known range of shockwave propagation speeds for multiphase fluids [36]. The low dominant frequencies correlate well with the calculated condensation front propagation frequencies for all bubble sizes and total void areas. However, the re-entrant motions are shown to primarily affect bubbles with sizes ranging between 3 μm and 7 μm. Finally, the P3 cavitation regime yields conclusions similar to P2, but with a crucial distinction: the difference between the void fraction fluctuations associated with the re-entrant jet and the total void fraction PSD is significantly reduced (the maximum difference in Strouhal numbers, max(∣St_f2_ − St_T2_∣)/St_T2_, is 1.0 in P2 and 0.43 in P3, respectively).

**Table 3:** Flow and void fraction mean features (l_c_, and U_t_), along with Strouhal numbers associated with f1 and f2 (St_f1_, St_f2_) in Fig. 8, Fig. 9, and T1 and T2 (St_T1_, St_T2_) in Fig. 10.

	$L_{c}$(mm)	$U_{t}$(m/s)	$A_{tot}$		$N_{b}3\mu m<D_{b}$		$N_{b}3\mu m<D_{b}<7\mu m$		$N_{b}D_{b}<7\mu m$		St_T1_	St_T2_
			St_f1_	St_f2_	St_f1_	St_f2_	St_f1_	St_f2_	St_f1_	St_f2_		
P1 Flow Condition	0.62	19.17	0.009	0.058	0.009	0.058	0.009	0.058	0.01	0.058	0.012	0.058
P2 Flow Condition	0.86	24.71	0.045	−	0.014	0.052	0.052	0.122	0.052	0.157	0.049	0.094
P3 Flow Condition	1.03	36.01	0.031	0.057	0.031	0.086	0.029	0.060	0.057	0.143	0.030	0.101

## 4 Conclusion

This study elucidated the distinct cavitating flow dynamics in micro-Venturi reactors with volumetric flow rates in the range of 0.1–0.2 ml/s across three pressure-driven regimes: transient detached (At 14.5 bar), periodic attached (at 17 bar), and fully developed cavitation (at 19 bar). Our findings reveal that residual bubbles, trapped in the boundary layer and reverse flow, are crucial for cavitation regeneration in the detached regime, triggered by interactions with transient shear-layer vortices. Bubble velocity analysis highlighted a size-dependent behavior influencing their transport. Increased upstream pressure is strongly associated with periodic attached cavitation with stable shedding frequencies, indicating a dominant, pressure-controlled mechanism. At the highest pressures, fully developed cavitation emerged, sustained by intense shear-layer activity and shear-induced breakup of larger bubbles, which then re-entered the separation zone, enhancing cavitation. These observations underscore the critical interplay of residual nuclei, vortex dynamics, and pressure in governing microscale cavitation in a Venturi, providing fundamental insights for optimizing microfluidic devices susceptible to cavitation. The results of this study are directly applicable to micro-scale devices, such as those used in silicon-based microfluidic systems, with microscale HC flows utilized in graphene exfoliation [37], drug delivery [38], and chemical processing, including advanced oxidation for wastewater treatment [15].

## Data availability

The data that support the findings of this study are available from the corresponding authors upon reasonable request.

## CRediT authorship contribution statement

**Mohammadamin Maleki:** Writing – original draft, Validation, Software, Methodology, Investigation, Formal analysis, Data curation. **Abhinav Priyadarshi:** Writing – original draft, Visualization, Investigation, Data curation. **Jolyon Cleaves:** Writing – original draft, Visualization, Resources. **Erçil Toyran:** Visualization. **Ali Kosar:** Writing – review & editing, Validation, Resources, Funding acquisition. **Iakovos Tzanakis:** Writing – review & editing, Writing – original draft, Visualization, Validation, Supervision, Resources, Project administration, Investigation, Funding acquisition, Conceptualization. **Morteza Ghorbani:** Writing – review & editing, Writing – original draft, Visualization, Validation, Supervision, Software, Resources, Project administration, Methodology, Investigation, Funding acquisition, Formal analysis, Data curation, Conceptualization.

## Declaration of competing interest

The authors declare the following financial interests/personal relationships which may be considered as potential competing interests: Morteza Ghorbani reports financial support was provided by TUBITAK (The Scientific and Technological Research Council of Turkey) BIDEB program, Project Code 123C216. Iakovos Tzanakis reports financial support was provided by Engineering and Physical Sciences Research Council. Morteza Ghorbani reports financial support was provided by The Royal Society. Morteza Ghorbani reports financial support was provided by British Council. If there are other authors, they declare that they have no known competing financial interests or personal relationships that could have appeared to influence the work reported in this paper.

## Figures and Tables

**Fig. B.2:**
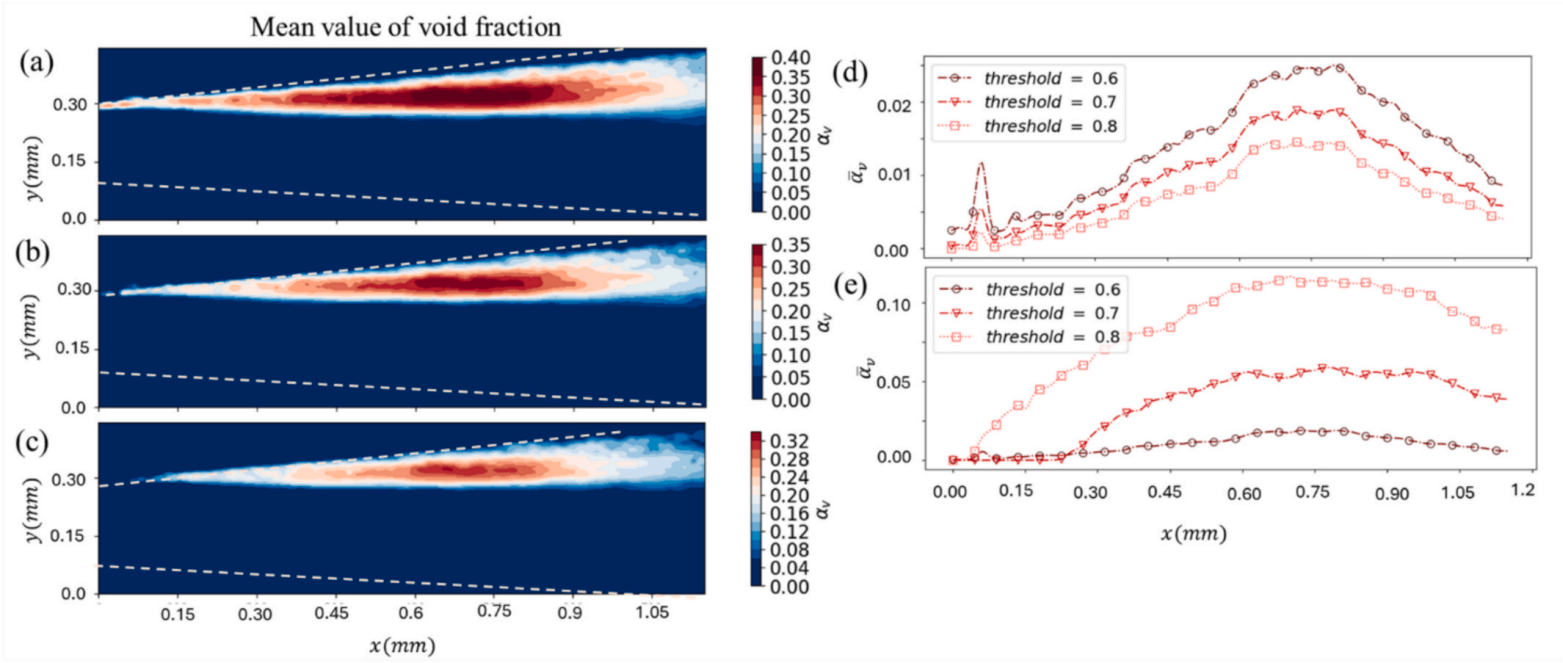
**.** Contours of mean (left column) and standard deviation (right column) of mean void fractions for P1 cavitation flow with binarization threshold of (a) 0.6, (b) 0.7, and (c) 0.8. (d) Comparison between of mean (time and y axis) void fractions of (e) P1 cavitation flow with different binarization threshold, and (e) different cavitation flows.

**Fig. B.3:**
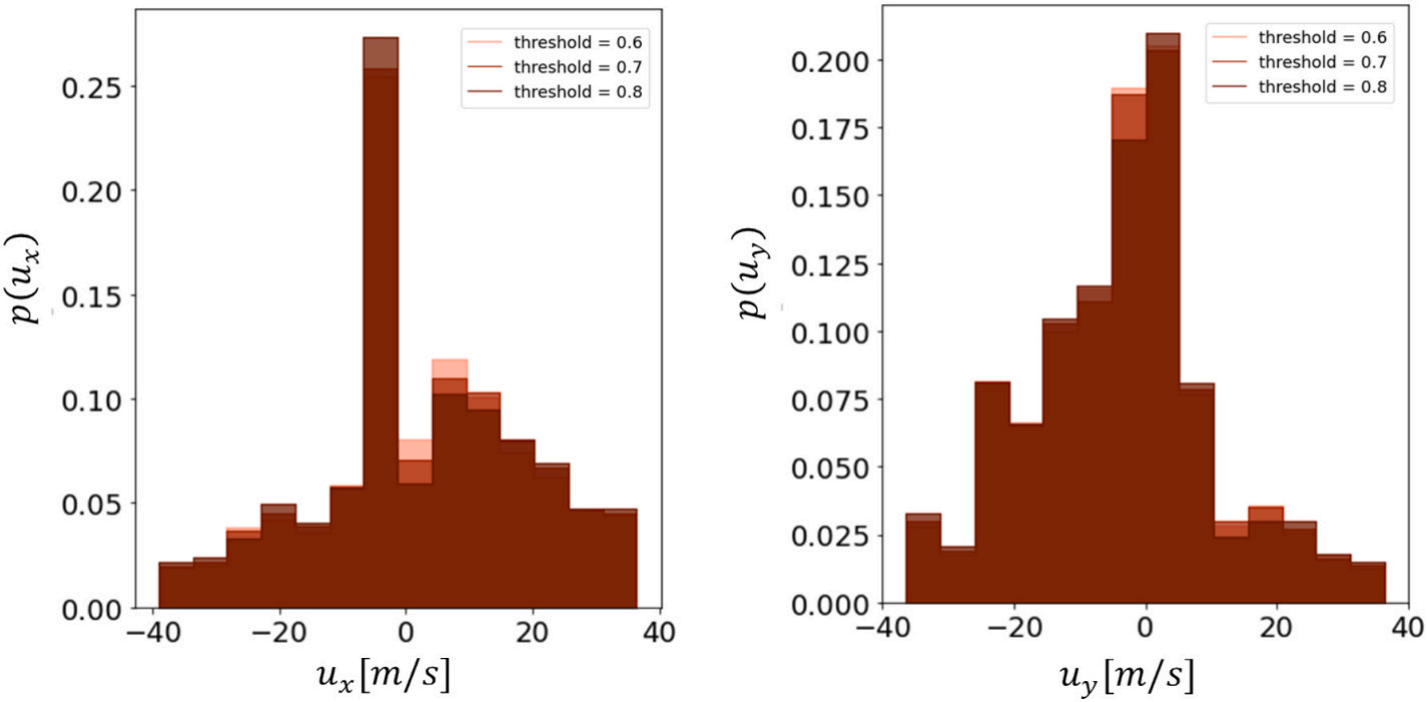
**.** Probability density functions (PDFs) of bubble velocity components (u_x_, u_y_) for the sensitive size range (D_b_ < 3 μm). The distributions are compared for three binarization thresholds: 0.6, 0.7 (nominal), and 0.8, demonstrating the systematic uncertainty on the velocity measurement.

**Fig. B.4:**
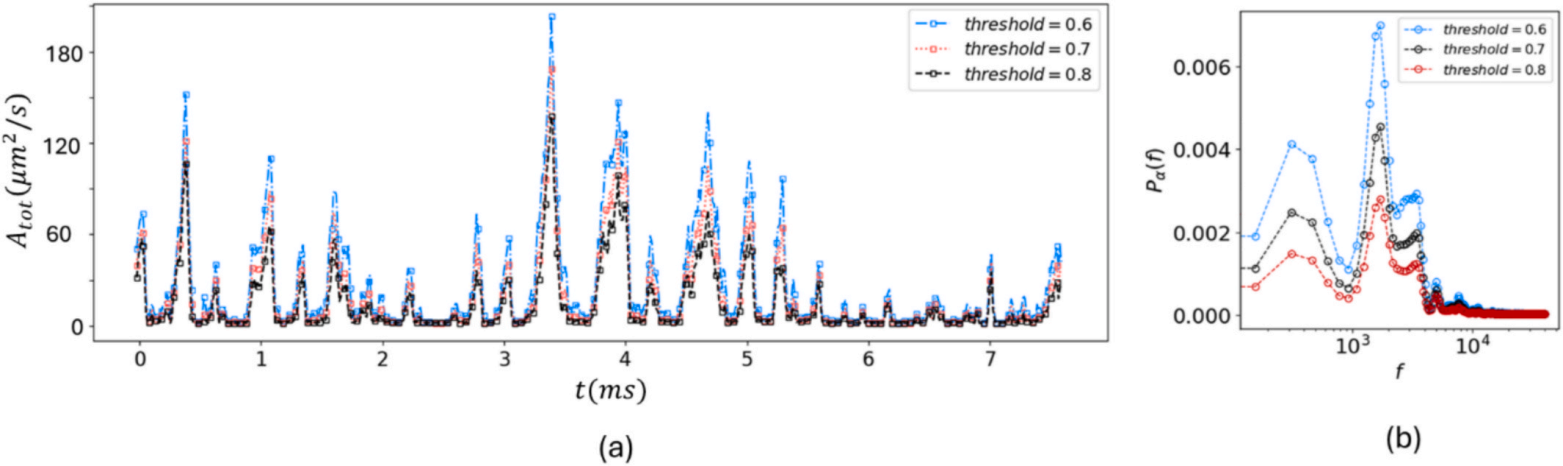
**.** Variation in total void fraction area (the left column) and corresponding spectral content (the right column) over time for P1 cavitation regime (t = 0 is the reference time). The distributions are compared for three binarization thresholds: 0.6, 0.7 (nominal), and 0.8, demonstrating the systematic uncertainty on the velocity measurement.

**Fig. B.1:**
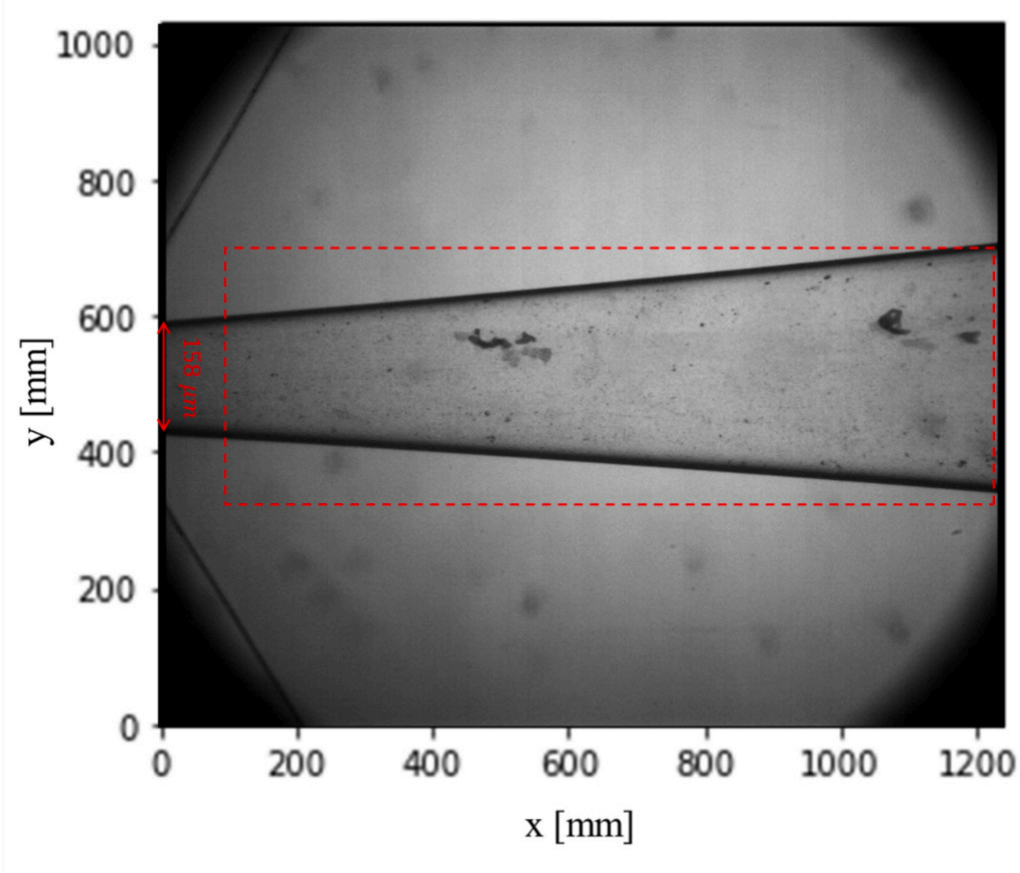
**.** Single snapshot from the high-speed camera results (924 x 768 pixels), along with estimated channel dimensions [µm] along the axes. Red box shows the cropped region used for image processing with resolution of 840 x 280 pixels.

**Fig. B.5:**
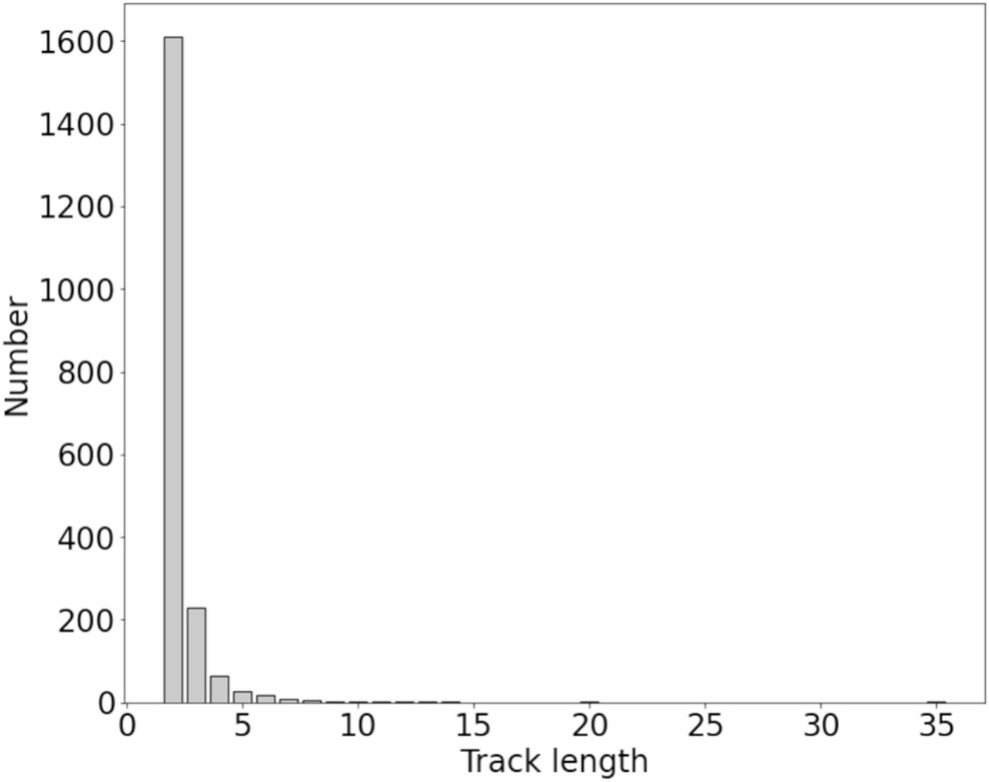
**.** distribution of the track length for 2 million fps visualizations results.

**Fig. B.6:**
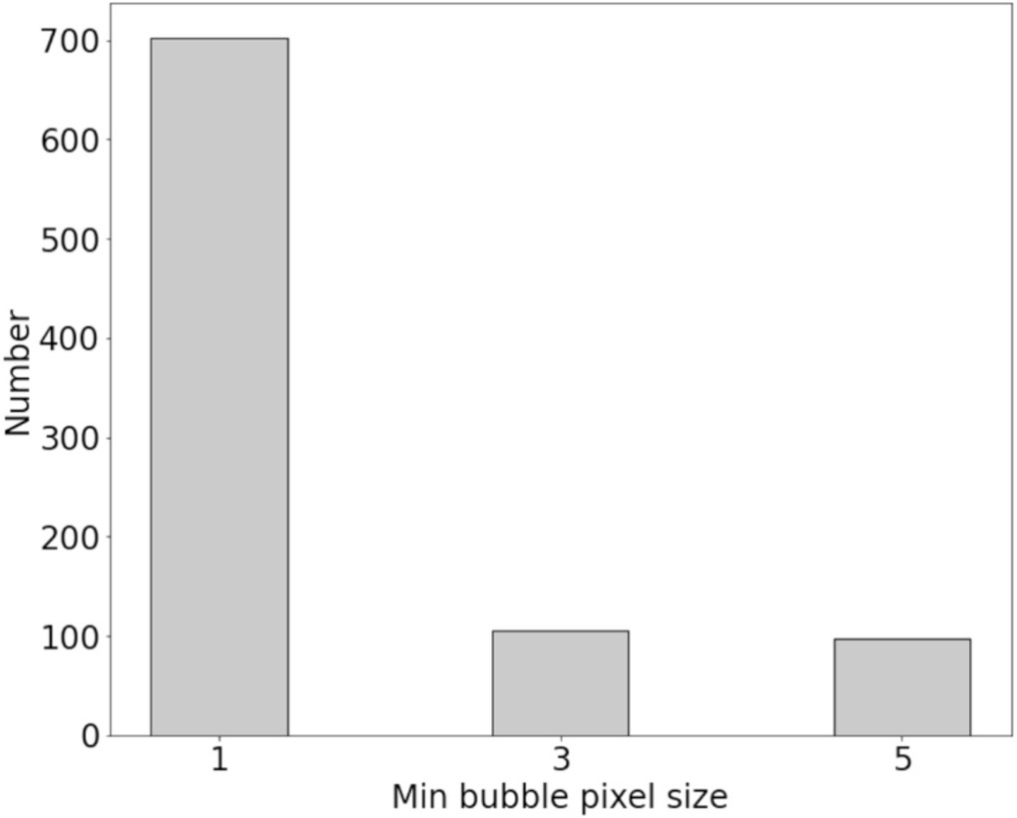
**.** distribution of the min pixel size for 2 million fps visualizations results.

**Fig. B.7:**
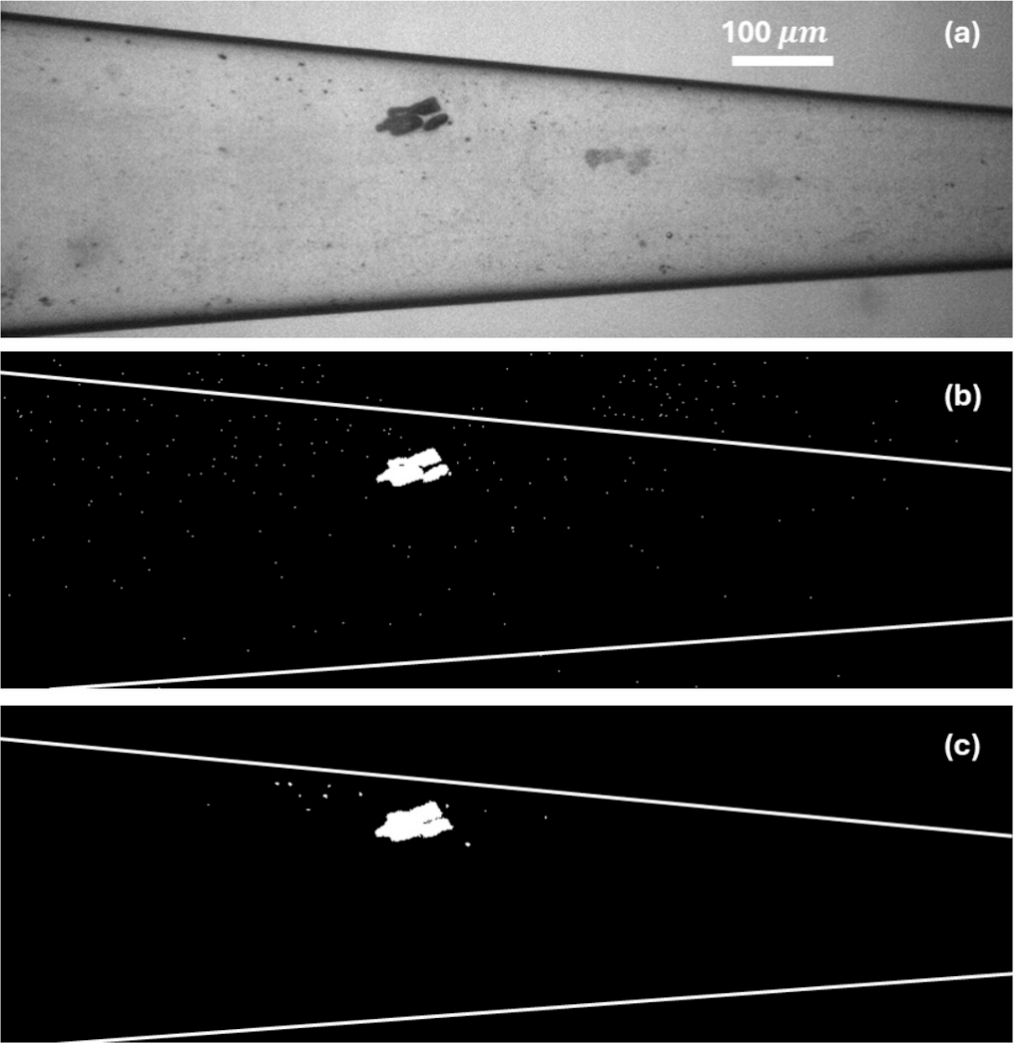
**.** Comparison of image segmentation techniques on a single snapshot of the venturi channel. (a) Original snapshot of the cavitating flow. (b) Corresponding segmented image using the Otsu global thresholding technique. (c) Corresponding segmented image using the Gaussian Mixture Model (GMM) adaptive thresholding technique.

## Appendix

### Appendix A . Data analysis

The bubble dynamics analysis comprised of two primary stages: image segmentation and bubble detection. For image segmentation, a Gaussian Mixture Model (GMM) was implemented using the scikit-learn library in the Python programming language[39]. The Gaussian Mixture Model (GMM) estimated the input data by modeling it as a multidimensional Gaussian mixture distribution. For a single input data point, the GMM calculated the probability of the input belonging to each cluster. The optimization of the GMM involved two key steps: the E-step, where the probability of the data point belonging to each cluster is estimated and the likelihood is evaluated based on the current parameters, and the M−step, where the Gaussian distributions of each cluster are updated to maximize the expected likelihood[39]. Before proceeding with the E-step and M−step, it was essential to determine the number of clusters and to initialize the covariance, mean, and weight parameters. Since there is a risk of missing the global optimal solution during likelihood maximization, multiple initializations were necessary.

First, preprocessing steps, such as subtracting the background from all images, were performed to enhance the data for segmentation. For each flow regime, a separate training and segmentation process was conducted using one-fifth of the snapshots. Three potential clusters were considered for each pixel. The initialization was performed using the k-means++ method. K-means provided a way to partition data points into N clusters. Once the points were assigned to clusters, the Gaussian mean and covariance for each cluster can be estimated. The k-means++ method builds on this approach: after selecting the first centroid randomly, the probability of choosing a subsequent data point as the next centroid was proportional to the square of its distance from the nearest existing centroid. This method strikes a balance between low initialization time and a reduced number of Gaussian Mixture iterations required for convergence. In contrast, random initialization typically required more iterations to converge. For this study, the maximum number of iterations, tolerance, and learning rate were set to 2000, 1e-6, and 1, respectively.

After segmentation (Fig. 2(a)), the pixels were categorized into two groups: the foreground, corresponding to the gas phase, and the background, corresponding to the liquid phase and the channel. Binary images were generated and analyzed to detect bubbles and calculate relevant parameters. The OpenCV Python library[40] was utilized for bubble tracking, and the results were used to determine various bubble characteristics and dynamics. Tracking and analyzing bubbles in cavitating flows presented several challenges, including bubble merging, breaking, collapsing, and emerging. To address these issues, the following processes were implemented: Bubble Identification: In each frame, a unique ID was assigned to each bubble. In sequential frames, the same ID was assigned to the bubble that is within a threshold distance and closest to the bubble in the previous frame. Bubble Break-up: When a bubble broke up, the resulting bubble with the larger area retained the original ID, while a new ID was assigned to the smaller bubble. Bubble Merging: When bubbles merged, the new bubble inherited the ID of the larger bubble, and the ID of the smaller bubble was no longer accounted for. Emergence and Disappearance: Newly emerging bubbles were assigned a new ID, while disappearing bubbles had their IDs deactivated. Additionally, a threshold area of more than three pixels (each frame has 840 × 280 pixels) was applied; detected foreground regions smaller than this threshold were disregarded. Furthermore, the threshold distance defined as the maximum allowable distance between two bubbles with the same ID in consecutive frames was set to 15 pixels. Given the spatial resolution of 1.34 μm/px at mean velocity the maximum jump is around 20.1 μm. Crucially, the dynamics of this study interest—namely, the upstream migration and evolution of residual bubbles within the low-momentum boundary layer is well resolved within the give temporal resolution (Fig. 5).

After bubble detection, a rectangular box (shown in Fig. 2(b) in yellow) and a blue plus sign were used to indicate the region occupied by each bubble and its centroid, respectively, in a given frame. Additionally, a red plus sign marked the center of mass of the bubbles. Each frame also provided detailed information per pixel, including time Index: the index of the snapshot; $A_{\mathrm{tot}}$: the total number of pixels occupied by all bubbles; $u_{x}$ and $u_{y}$: the velocity of each bubble in the x and y directions, determined by the pixel displacement compared to the previous time step. The aforementioned quantities were converted to SI units to extract the following parameters for each time step (t):

(i) Number of detected bubbles:$N\left(t\right).$
(ii) Area of each detected bubble:$A_{b,}\left(t\right)\left[m^{2}\right].$
(iii) Position of each bubble centroid: $x_{b}\left(t\right)\left[m\right]=\left[x_{b},y_{b}\right]$
(iv) velocity of each bubble centroid: $u_{b}\left[m/s\right]=\left[{\delta x}_{b}/\delta t,{\delta y}_{b}/\delta t\right]=\left[{u_{x}}_{b},{u_{y}}_{b}\right]$

The parameters defined above (where *b* denotes the bubble index) were used to derive additional quantities, including, the number of bubbles falling into specific size ranges, the mean spatial coordinates of bubbles, the mean velocity of bubbles.

To analyze the frequency characteristics of cavitation regimes, the pre-multiplied power spectral density (PSD) was calculated for both the number of bubbles in specific size ranges and the total bubble area. For all cases, the sampling interval was *T* = 7.7 ms, corresponding to a sampling rate of fs = 8 × 10^4^ Hz. The analysis utilized four-time segments, each with a length of 256 samples (equivalent to 3.2 ms), applying a Hanning window and a 50 % overlap (128 samples) between segments to minimize spectral leakage and improve statistical averaging. This configuration yields a frequency resolution of approximately 312.5 Hz and allows for analysis up to the Nyquist frequency of 40 kHz [41]. The primary temporal resolution used for long-term flow characterization and frequency analysis was set at fs = 80,000 fps. This sampling rate establishes a Nyquist frequency of 40 kHz. The highest dominant frequency measured in the PSD analysis, corresponding to the re-entrant jet dynamics, was found to be around 14 kHz (see Fig. 10). This ensures a Nyquist margin of approximately 2.85× (40 kHz/14 kHz), which is considered sufficient for accurately resolving the flow's dominant temporal dynamics and providing a robust safeguard against aliasing of the bulk cavitation motions. While ultra-high-speed imaging was used to study micro-scale bubble events, the 80,000 fps rate is confirmed as adequate for accurately resolving the macro-scale void fraction dynamics and shedding frequencies.

## Appendix B . Uncertainty

There are several uncertainties that may contribute to the errors in the extracted data. Some of them are detailed as follows:

## Impact of camera resolution

Fig. B.1. represents a single shot of the high-speed camera results along with horizontal and vertical dimensions along the axes (extrapolated based on throat dimension). The setting yields the resolution of a = 1.34 μm/pixel, which is appropriate for capturing void fractions with width than 1.34 μm.

## Errors related to the 3D out-of-plane motion of a bubble where a bubble can move out of focus, coincide and overlap

The minimal channel depth effectively mitigates out-of-plane errors. This is achieved, first, by restricting the overlap of bubbles—particularly for characteristic bubble lengths smaller than 30 μm (half of channel depth)—and second, by ensuring the entire channel remains within the focal plane of the imaging system, thus eliminating errors caused by out-of-focus blurring.

## Presence of noises that can lead to false detection

To mitigate the presence of noise and false detections, two distinct procedures were implemented. First, background subtraction was performed during the segmentation process to effectively remove stationary defects or spatial non-uniformities within the imaging domain. Second, a minimum size threshold was applied: particles smaller than 3 pixels were disregarded to filter out small impurities and image noise. The details on the effect of the selected minimum bubble size on important parameters are further discussed in the following sections (Fig. B.6).

## Parameter settings in image segmentation and voids detection

The results (mean void values, velocity distribution, PSD values) for three different threshold values were examined to ensure about the robustness of the method (Fig. B.2, Fig. B.3, Fig. B.4). In addition, comparison between Otsu and GMM results (Fig. B.7) shows the superior performance of GMM.

To validate the reliability of the image processing, the void segmentation and bubble tracking algorithms were quantitatively assessed against 50 manually labeled frames for each flow regime (P1, P2, and P3). The results confirm high accuracy, with Precision (true positives over total positive predictions) recorded at 0.941 (P1), 0.977 (P2), and 1.0 (P3). This demonstrates a negligible false detection rate in the identified bubble population. Recall (true positives over total actual positives) was maintained at acceptable levels (0.903, 0.754, and 0.735), with missing detections primarily attributed to motion blur leading to ambiguity in segmentation. Given the objective of analyzing reliable bubble dynamics, precision was prioritized. To ensure the robustness of the derived trajectories, the analysis was restricted to tracks with a minimum duration of two sequential frames to filter out transient noise.

To assess the robustness of our quantitative results against image processing parameters, the systematic uncertainty associated with the binarization threshold was propagated across a range of 0.6 to 0.8 based on the nominal setting. This evaluation was performed on the P1 cavitation regime, which presents the most challenging and sensitive case due to its dispersed bubble structure. The analysis focused on the resultant deviations in: 1) the mean void fraction distribution, 2) the velocity distribution (u_x_ and u_y_) of the smallest and most sensitive bubble fraction (D_b_ < 3 μm), and 3) the spectral characteristics of the total void area. The maximum systematic deviations observed between the nominal threshold result and the bounds (0.6 or 0.8) are summarized in Fig. B.2, Fig. B.3, Fig. B.4. The maximum deviation in the time-averaged void fraction (${\overset{¯}{\alpha }}_{v}$) was found to be approximately 0.0067 (Fig. B.2d). For the velocity distributions (Fig. B.3), the maximum deviations recorded were 0.012 m/s for u_x_ and 0.019 m/s for u_y_. Spectral analysis showed a maximum PSD deviation of approximately 0.0023 (Fig. B.4). The stability of the distribution morphology across this threshold range indicates that the systematic uncertainty associated with binarization does not affect the main conclusions drawn when comparing the distinct cavitation regimes.

The track length distribution for the analysis utilizing the 2 million fps visualizations is presented in Fig. B.5. As previously discussed, track lengths shorter than two frames were excluded from the analysis to minimize the inclusion of noise. The results consistently show an exponential decrease in the number of tracks as the track length increases, which is characteristic of transient bubble events.

Fig. B.6. represents distribution of the min pixel size for 2 million fps visualizations results. As it is observed the changes in number of stationary bubbles (bubbles with absolute velocity of zero), achieved to a nearly unform value after passing the threshold of 3 pixels. Assuming a 1 pixel × 3 pixel area for the strictest case, the threshold area is 4.02  μm^2.^

Comparison of image segmentation techniques on a single snapshot is provided in Fig. B.7. The preliminary visual comparison demonstrated that Otsu's method (Fig. B.7) resulted in significant over-segmentation, particularly in the vicinity of the channel walls and far-field regions outside the cavitation zone. This led to a substantial increase in false positives, especially for smaller objects (noise) and artifacts caused by reflections. Consequently, we are confident that using Otsu's method would compromise the key quantitative results, such as the size-dependent velocity distributions and dominant frequency bands.

While a full statistical comparison showing the robustness of our key results (size-dependent velocities and frequency bands) across both methods was not systematically performed due to the immediately apparent failure of Otsu's method to segment the images meaningfully.
